# Knowledge, attitude and compliance of infection control guidelines among dental faculty members and students in KSU

**DOI:** 10.1186/s12903-018-0706-0

**Published:** 2019-01-09

**Authors:** Ghada Alharbi, Noura Shono, Lamya Alballaa, Alaa Aloufi

**Affiliations:** 1 Princess Noura University, Riyadh, Kingdom of Saudi Arabia; 2 King Saud University, Riyadh, Kingdom of Saudi Arabia; 3 King Saud Medical City, Riyadh, Kingdom of Saudi Arabia; 4 Taibah University, Madinah, Kingdom of Saudi Arabia

**Keywords:** Infection control, Knowledge, Attitude, Compliance, Dental student, Faculty members

## Abstract

**Background:**

Infection is one of the most crucial problems in health care services worldwide. It is considered one of the most important causes of morbidity and mortality associated with clinical, diagnostic and therapeutic procedures. Therefore, the purpose of this study was to investigate knowledge, attitude, and compliance with recommended infection control guidelines among dental faculty members and students at King Saud University, Riyadh, Kingdom of Saudi Arabia.

**Methods:**

A cross-sectional study was conducted to obtain information regarding knowledge, attitude, and compliance with recommended infection control guidelines. The sample (*n* = 317) comprised of dental faculty members and students (3rd, 4th and 5th year) in both male and female campuses of College of Dentistry (KSU).

This questionnaire contained three parts (knowledge, attitude, and compliance) and was distributed to the participants. After validation of the survey, data were collected, entered and analyzed by SPSS software.

**Results:**

A total of 317 dental faculty members and students participated in this study. Out of the total study subjects, 141 (44.5%) were female and 176 (55.5%) were male. A comparison between dental faculty members and students was made based on their knowledge, attitude, and compliance, which resulted in almost equal percentages of knowledge (49.6, 49.0% respectively). In addition, it revealed that faculty members’ attitude toward infection control in the dental clinic was more positive compared to their compliance with the infection control guidelines (70.6, 65.2% respectively) while with the students it was vice versa (67.2, 69.6% respectively).

There is no statistically significant difference in the knowledge and attitude of dental faculty members and students regarding infection control guidelines (*P* > 0.05).

**Conclusion:**

Our study showed that dental undergraduate student and faculty members at KSU demonstrated a good adherence to infection control guidelines. On the other hand, there was a lack in the knowledge of the basics of infection control standards.

## Background

Knowledge, attitude, and compliance act as three key elements, which make up the dynamic system of life itself. Knowledge is defined as information that could be acquired through various ways namely reading, experience and comprehension. Furthermore, it is the basic criterion that allows one to differentiate between right and wrong. On the other hand, according to the English dictionary attitude refers to the manner, feeling or position, with regard to a person or thing; tendency or orientation, especially of the mind. While compliance is the reflection of rules and knowledge that leads to action. Thus, right knowledge, positive attitude, and good compliance are imperative to guide health care professionals in treating and serving their patients [1].

Infection is one of the most crucial problems in health care services worldwide. It is considered one of the most important causes of morbidity and mortality associated with clinical, diagnostic and therapeutic procedures [2]. Infection control is defined as “Measures practiced by health care personnel to reduce the risks of transmission of infectious agents to patients and employees (e.g. proper hand hygiene, scrupulous work practices, use of personal protective equipment (PPE), such as masks or respirators, gloves, gowns and eye-protection)” (Centers for Diseases Control and Prevention 2005). Infection control measures include contact, droplet and airborne precautions based on how an infectious agent is transmitted [3]. In general, health care workers that do not use proper infection control procedures while providing patient care are more susceptible to infectious diseases [4]. A paper written by Laheij et al., in 2012, evaluated the literature to determine the risk of infection and cross-transmission by bacteria and viruses and that are of particular relevance in the dental practice environment (e.g. Hepatitis B, C and D viruses, HSV, VZV and HIV). The paper concluded that the transmission of, and infection with, Hepatitis B virus poses the greatest risk for both the dental team and the patients. However, the literature on the transmission of the other viruses and bacteria is scarce and the risk for transmission resulting in an infection with these microorganisms seems low [5].

During dental procedures, transmission of infections could occur either through direct contact with blood, saliva or contaminated treatment water from dental units, injury with an anesthetic needle or splash exposure of the mucous membranes, droplets, and aerosols or indirect contact with contaminated instruments and surfaces. By using safety precautions at work and implementing infection control guidelines, accidental exposure to infections in dental settings can be avoided [6, 7].

In the late 1970s, a study found that dentists were three times more likely than the general population to be infected by hepatitis B [8]. In the United States of America, the United States Department of Labor’s Occupational Safety & Health Administration (OSHA), in 1991, issued the Blood-borne Pathogens Standard developed to protect workers from the risk of blood-borne pathogens exposure such as Hepatitis B, Hepatitis C, and HIV/AIDS [9]. However, the limitations of universal precautions were subsequently recognized and in 1996, the CDC adopted the term “standard precautions” to apply a broader concept of prevention and transmission of infectious diseases. Standard precautions integrate and expand the elements of universal precautions into a standard of care designed to protect health care professionals and patients from pathogens in hospital settings [10]. The CDC new recommendation is that every dental clinic must have an infection prevention coordinator. The coordinator is responsible for the development of a written infection prevention policies based on the CDC’s evidence-based guidance in the updated resource. The coordinator should assist the others in the clinic so they are up to date with the supplies and equipment necessary to ensure infection prevention [11].

Because of the limitations of routine health history information, the application of standard precautions to all patients becomes necessary. As some patients visiting dental clinics appear to be healthy, with normal physical examination findings and medical histories, the application of standard precautions should not be based on patients’ appearance. By implementing infection control guidelines in addition to vaccinations and proper post-exposure management, exposure to infections in dental settings can be prevented [12].

According to Hazelkorn HM study in 1989, dentists apparently know what to do to protect themselves from contamination. Nevertheless, very few dentists discussed AIDS or HIV while recording a pretreatment history even if the patient was perceived to be in a high-risk group [13]. Strategies to protect health workers include 1-implementation of standard precautions, 2-immunization against infectious diseases of concern,3- provision of personal protective equipment,4- correct cleaning and disinfection of surfaces and equipment to remove pathogens, 5- sterilization of instruments and 6- proper techniques for handling sharp instruments and the management of exposure that are recommended by WHO [14]. Dental education can play an important role in dental students training, helping them to adopt adequate knowledge and attitude related to infection control [15].

In the College of Dentistry at King Saud University, infection control lectures start as early as the 1st year. Infection control procedures are applied in dental laboratories in the 2nd and 3rd years. In the 4th and 5th years of study, students apply the concepts of infection control in the clinical training sessions. The Personal Protective Equipment (PPE) in dental clinics of King Saud University includes disposable gowns, masks, gloves and face shields. These are to be used at all times when treating a patient.

Any violation of infection control guidelines will be detected from the overall clinical evaluation of the students. Clinical director and supervisors are emphasizing and monitoring the using of PPE by students and wearing protective goggles by patients. Students are restricted from wearing jewelry in the dental clinics and must have short fingernails and not nail polished. They are also required to have taken the HB vaccination before entering the clinics.

The purpose of this study is to investigate knowledge; attitude and compliance with recommended infection control guidelines among faculty members, 3rd, 4th and 5th-year dental students at the College of Dentistry King Saud University, Riyadh, Kingdom of Saudi Arabia.

## Methods

A cross-sectional study was conducted among dental faculty members and students (3rd, 4th and 5th year) in both male and female campuses of College of Dentistry, King Saud University, Riyadh city, Kingdom of Saudi Arabia. A questionnaire was designed to obtain information about infection control knowledge, attitude, and compliance.

The questionnaire was pretested via a pilot study and it was validated. The pilot study was conducted to a small group of faculty members and students to assess the validity, time is taken to fill up the questions, common understanding and interpretation of the question by the respondents. Forty surveys were distributed on October 2014, 5 surveys for each sample group. Based on participants’ feedbacks, some changes in the survey format were made for example: correcting language and grammar mistakes, summarizing and shortening long questions and omitting some questions because of repetition. The participants of the pilot study were excluded from the main study. The dental faculty members and students who had been included in the main study have voluntarily participated in the survey. The final survey was conducted between November 2014 and February 2015. The questionnaire was framed with the help of experts in the field and it kept the sample group in mind. A self-administrated questionnaire consisting of 25 close-ended questions was used for data collection. The dental students were given the questionnaire in the classrooms and asked to fill it out in 10 min. The faculty members were given the same questionnaire in various departments.

In the study survey, no personally identifiable information captured. Therefore, responses cannot be traced back to the respondents and the data were anonymous. The surveys were distributed and collected in sealed envelopes to assure confidentiality.

Upon the directions of the College of Dentistry Research Center (CDRC) in KSU, no ethical approval was requested to start the study. It only involved asking students and faculty members of King Saud University non-sensitive questions that are strictly within their professional competence using surveys. Moreover, the data collected is not personally identifiable and used solely for the purpose of this study.

Regarding the consent to participate, it was stated at the front page of the survey distributed that by completing the survey, the participants would give their consent to be part of the study.

There were 7 questions to assess knowledge, 7 questions to assess attitude, and 11 questions to judge infection control compliance of the respondents. The questionnaire collected data on knowledge pertaining to infection control procedures, sterilization, disinfection of instruments, occupational hazards and immunization. Participants who were present on the days of the survey were included; no attempt was made to further invite the faculty members and students absent during the survey days. The subjects who did not fill the questionnaire completely were excluded.

### Statistical analysis

Questionnaire data were entered into the Statistical Package for Social Sciences SPSS® software for Windows® ver.21. Analysis of variance (ANOVA) was used to compare the mean of knowledge, attitudes, and compliance scores while independent t-test was used to compare whether the two sample groups have different average values. The level of significance was set at *p* < 0.05 for all statistical tests.

## Results

**Fig. 1:**
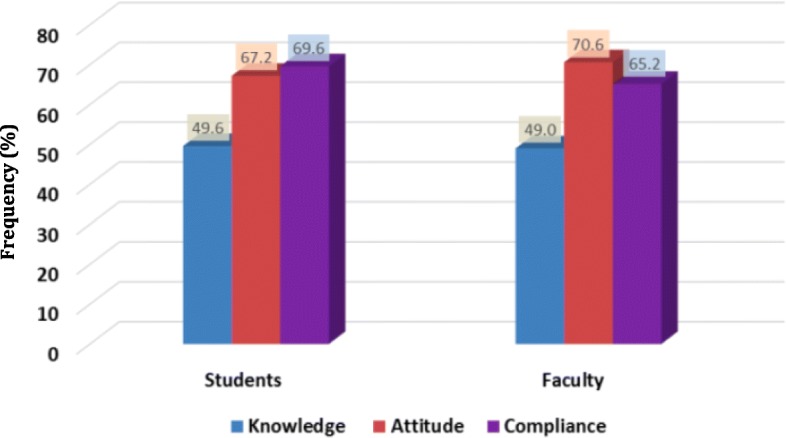
The Knowledge, attitude and compliance of faculty members and dental students regarding infection control guidelines by percentage

A total of 317 students and faculty members participated in this study. The response rate for this study was as following; out of 191 faculty members, only 110 responded to our survey with 20 participants dropped out and 90 surveys were included. Out of the total number of dental students (which is 317), only 277 responded to our survey with 50 dropped out, as they haven’t completed the survey and 227 were included. The subjects who were absent or on leave or on an abroad scholarship were excluded too. Table 1 shows the detailed sample size table outlining the number of students in different under graduation year level that participated in our study. Table 2 shows the number of faculty members with their years of experience. Out of the total study subjects, 141 (44.5%) were female and 176 (55.5%) were male. A comparison between dental faculty members and students was made based on their knowledge, attitude, and compliance, which resulted in almost equal percentages of knowledge (49.6 and 49.0%) respectively (Fig. 1). There is no significant difference in the knowledge of dental faculty members and students regarding infection control guidelines (*p* > 0.05). However, faculty members’ knowledge about the proper type of solution for washing their hands in the dental clinic (antiseptic solution) was significantly higher than that of the students (*P*=. 003).

**Table 1:** Demographics data of dental students’ participants

Demographics	3rd year	4th year	5th year
	(*n* = 80)	(*n* = 62)	(*n* = 85)
Age	Under 26	Under 26	Under 26
Gender (F/M)	(30/50)	(30/32)	(33/52)
Total	227

**Table 2:** Demographics data of dental faculty participants

Demographics		n (%)
Age	< 26 years old	5 (5.6%)
	26–35 years old	45 (50%)
	36–45 years old	25 (27.8%)
	> 46 years old	15 (16.7%)
Gender (F/M)		48/42
Years of experience	0–5 years	29(32.2%)
	6–10 years	33 (36.7%)
	11–15 years	10 (11.1%)
	16–20 years	8 (8.9%)
	> 21 years	10 (11.1%)

The majority of the students (84.1%) consider heat sterilization (Autoclaving) as the most reliable method (Table 3). About 21.6% had been pricked by a sharp instrument while treating patients. Surprisingly, almost half of them (55.1%) went to the hospital to be tested for HBV (Table 4). There was a significant difference in needle stick injury occurrence among the dental students (*p* = .007) with fifth-year students showing the highest percentage of needle stick injuries (Table 5). Almost all female and male students wear and change gloves between patients that accounted for 95.4% (Table 6).

**Table 3:** The knowledge of dental faculty members and students regarding infection control guidelines

Questions	M&F Students		M&F Faculty		M&F Total		
	Correct	Incorrect	Correct	Incorrect	Correct	Incorrect	*P* value
Q1. The aim of sterilization is the destruction of:	192(84.6%)	35(15.4%)	69(76.7%)	21(23.3%)	261 (82.3%)	56 (17.7%)	.122
Q2. Most reliable method sterilization is:	191 (84.1%)	36 (15.9%)	78 (86.7%)	12 (13.3%)	269 (84.9%)	48 (15.1%)	.573
Q3. Minimum time required for sterilization in autoclave is:	102 (44.9%)	125 (55.1%)	49 (54.4%)	41 (45.6%)	151 (47.6%)	166 (52.4%)	.127
Q4. The temperature for sterilization in autoclave is:	108 (47.6%)	119 (52.4%)	36 (40%)	54 (60%)	144 (45.4%)	173 (54.6%)	.221
Q5. Which of the following has the highest rate of transmission via saliva:	83 (36.6%)	144 (63.4%)	33 (36.7%)	57 (63.3%)	116 (36.6%)	201 (63.4%)	.986
Q6. What immediate action should be taken in case of direct blood contact with an HIV patient:	85 (37.4%)	142 (62.6%)	41 (45.6%)	49 (54.4%)	126 (39.7%)	191 (60.3%)	.192
Q7. What do you use to wash your hands?	124 (54.6%)	103 (45.4%)	65 (72.2%)	25 (27.8%)	189 (59.6%)	128 (40.3%)	.003

*M* Male, *F* Female

**Table 4:** The attitude of dental faculty members and students regarding infection control guidelines

Questions	M&F Students		M&F Faculty		M&F Total		
	Yes	No	Yes	No	Yes	No	*P* value
Do you prefer oral mouth rinse before the commencement of any treatment procedure?	125 (55.1%)	102 (44.9%)	59 (65.6%)	31 (34.4%)	184 (58.0%)	133 (42.0%)	.084
Do you think isolation is important in infection control?	222 (97.8%)	5 (2.2%)	89 (98.9%)	1 (1.1%)	311 (98.1%)	6 (1.9%)	.522
Do you wash your hands after examination?	214 (94.3%)	13 (5.7%)	87 (96.7%)	3 (3.3%)	301 (95.0%)	16 (5.0%)	.382
Is disinfection of the dental chair, clinic and dental office, required between patients?	219 (96.5%)	8 (3.5%)	88 (97.8%)	2 (2.2%)	307 (96.8%)	10 (3.2%)	.551
Did you receive HBV Immunization Vaccine?	212 (93.4%)	15 (6.6%)	83 (92.2%)	7 (7.8%)	295 (93.1%)	22 (6.9%)	.713
Did you prick your skin with a sharp instrument while treating a patient?	49 (21.6%)	178 (78.4%)	23 (25.6%)	67 (74.4%)	72 (22.7%)	245 (77.3%)	.448
-If yes, Did you go to the hospital and get tested for HBV?	27 (55.1%)	22 (44.9%)	16 (69.6%)	7 (30.4%)	43 (59.7%)	29 (40.3%)	.202

*M* Male, *F* Female

**Table 5:** The attitude of dental students regarding infection control guidelines

Questions	M&F 3rd year		M&F 4th year		M&F 5th year		
	Yes	No	Yes	No	Yes	No	*P* value
Do you prefer oral mouth rinse before the commencement of any treatment procedure?	59 (73.8%)	21 (26.3%)	25 (40.3%)	37 (59.7%)	41 (48.2%)	44 (51.8%)	.000
Do you think isolation is important in infection control?	77 (96.3%)	3 (3.8%)	60 (96.8%)	2 (3.2%)	85 (100%)	0 (0%)	.214
Do you wash your hands after examination?	72 (90%)	8 (10%)	58 (93.5%)	4 (6.5%)	84 (98.8%)	1 (1.2%)	.049
Is disinfection of the dental chair, clinic and dental office, required between patients?	76 (95%)	4 (5%)	59 (95.2%)	3 (4.8%)	84 (98.8%)	1 (1.2%)	.335
Did you receive HBV Immunization Vaccine?	75 (93.8%)	5 (6.3%)	55 (88.7%)	7 (11.3%)	82 (96.5%)	3 (3.5%)	.173
Did you prick your skin with a sharp instrument while treating a patient?	8 (10%)	72 (90%)	17 (27.4%)	45 (72.6%)	24 (28.2%)	61 (71.8%)	.007
-If yes, did you go to the hospital and get tested for HBV?	2 (25%)	6 (75%)	6 (35.3%)	11 (64.7%)	19 (79.2%)	5 (20.8%)	.000

*M* Male, *F* Female

**Table 6:** The compliance of dental students regarding infection control guidelines

M&F Students					
Questions	Always	Often	Sometimes	Rarely	Never
Wash your hands prior to wearing gloves	71 (31.3%)	68 (30%)	48 (21.1%)	26 (11.5%)	14 (6.2%)
Wear gloves during treatment	216 (95.2%)	4 (1.8%)	3 (1.3%)	0 (0%)	4 (1.8%)
Change gloves between patients	217 (95.6%)	4 (1.8%)	2 (0.9%)	0 (0%)	4 (1.8%)
Wear face mask during treatment	206 (90.7%)	13 (5.7%)	4 (1.8%)	1 (0.4%)	3 (1.3%)
Change face mask between patients	166 (73.1%)	32 (14.1%)	14 (6.2%)	6 (2.6%)	9 (4%)
Wear a protective face shield during treatment	105 (46.3%)	41 (18.1%)	36 (15.9%)	27 (11.9%)	18 (7.9%)
Disinfect face shield between patients	117 (51.5%)	28 (12.3%)	30 (13.2%)	24 (10.6%)	28 (12.3%)
Wear a protective gown during treatment	209 (92.1%)	8 (3.5%)	4 (1.8%)	2 (0.9%)	4 (1.8%)
Change protective gown between patients	154 (67.8%)	30 (13.2%)	22 (9.7%)	11 (4.8%)	10 (4.4%)
Ask the patient to wear protective eyewear during treatment	140 (61.7%)	42 (18.5%)	31 (13.7%)	10 (4.4%)	4 (1.8%)
Use of rubber dam	137 (60.4%)	58 (25.6%)	23 (10.1%)	3 (1.3%)	6 (2.6%)

**Fig. 2:**
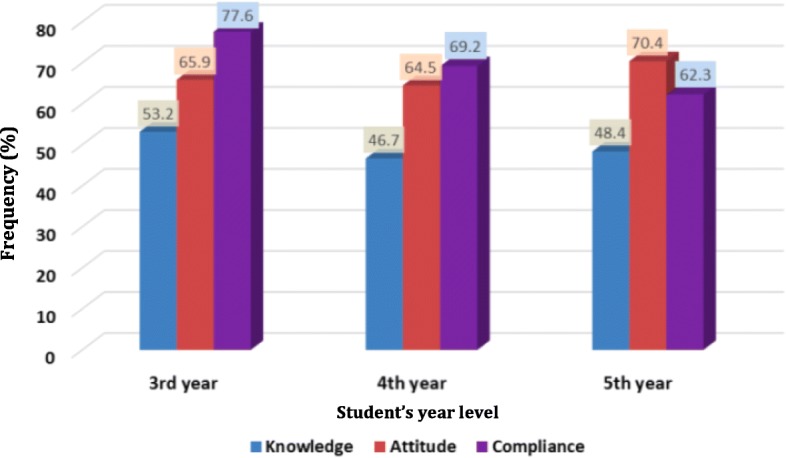
The Knowledge, attitude and compliance of dental students regarding infection control guidelines by percentage

Upon comparing 3rd, 4th and 5th-year students regarding their knowledge, attitude, and compliance, it was found that 3rd-year students have the highest level of knowledge and compliance (53.2 and 77.6%) respectively. On the other hand, 5th-year students showed the most positive attitude (70.4%) as illustrated in (Fig. 2).

Regarding faculty members, 76.7% reported that the aim of sterilization is the destruction of all microorganisms (both spore and non-spore forming). A small percentage of the faculty (36.7%) thought that Tuberculosis has the highest rate of transmission via saliva as shown in (Table 3). The results revealed that the percentage of faculty members who prefer the use of oral mouth rinse for their patients before treatment and believe in the importance of isolation in infection control were 65.6 and 98.9%, respectively (Table 4).

Regarding the use of rubber dam almost equal percentages were found in two opposite scales of compliance, in which 34.4% of clinicians always use it while 26.7% never do (Table 7). There was no significant difference found in the attitude toward infection control guidelines between faculty members and dental students at the College Of Dentistry In King Saud University (*p* > 0.05). In addition, it revealed that faculty members’ attitude toward infection control in the dental clinic was more positive compared to their compliance with the infection control guidelines while with the students it was vice versa (Fig. 1).

**Table 7:** The compliance of dental faculty members regarding infection control guidelines

M&F Faculty					
Questions	Always	Often	Sometimes	Rarely	Never
Wash your hands prior to wearing gloves	47 (52.2%)	25 (27.8%)	8 (8.9%)	5 (5.6%)	5 (5.6%)
Wear gloves during treatment	87 (96.7%)	3 (3.3%)	0 (0%)	0 (0%)	0 (0%)
Change gloves between patients	89 (98.9%)	1 (1.1%)	0 (0%)	0 (0%)	0 (0%)
Wear face mask during treatment	83 (92.2%)	6 (6.7%)	0 (0%)	1 (1.1%)	0 (0%)
Change face mask between patients	58 (64.4%)	11 (12.2%)	12 (13.3%)	7 (7.8%)	2 (2.2%)
Wear a protective face shield during treatment	49 (54.4%)	17 (18.9%)	14 (15.6%)	9 (10.0%)	1 (1.1%)
Disinfect face shield between patients	47 (52.2%)	15 (16.7%)	15 (16.7%)	11 (12.2%)	2 (2.2%)
Wear a protective gown during treatment	63 (70%)	12 (13.3%)	7 (7.8%)	6 (6.7%)	2 (2.2%)
Change protective gown between patients	44 (48.9%)	11 (12.2%)	19 (21.1%)	12 (13.3%)	4 (4.4%)
Ask the patient to wear protective eyewear during treatment	48 (53.3%)	21(23.3%)	14 (15.6%)	5 (5.6%)	2 (2.2%)
Use of rubber dam	31 (34.4%)	7 (7.8%)	18 (20.0%)	10 (11.1%)	24 (26.7%)

*M* Male, *F* Female

**Fig. 3:**
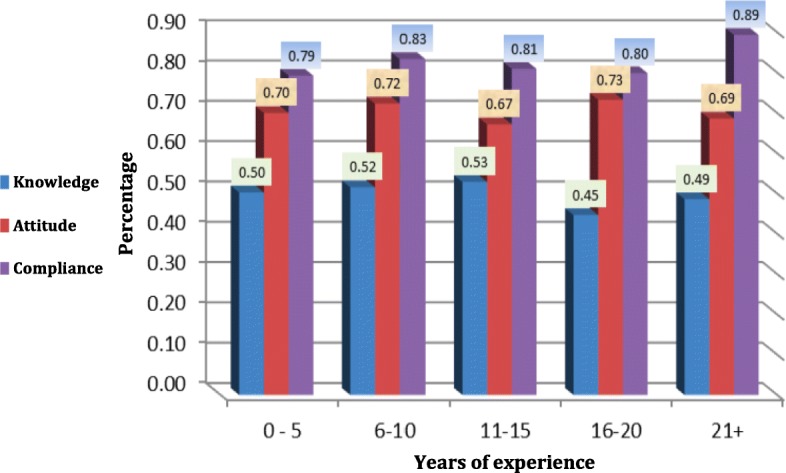
The Knowledge, attitude and compliance of dental faculty members regarding infection control guidelines according to years of experience

The study showed that the most knowledgeable faculty members are the ones with 11–15 years of experience. Positive attitude toward infection control in the dental clinic was mostly noticed in the members who had 16–20 years of experience in the field. It was found that dental professionals with more than 21 years of experience have the highest compliance with CDC infection control guidelines (Fig. 3).

## Discussion

Our study showed an overall good adherence to standard isolation precautions among dental faculty members and students in KSU. While the attitude and compliance levels were acceptable, the knowledge was fair. The deficit of knowledge could be due to the inadequacy of infection control educational materials during years of study. Another reason might be the lack of belief that practice of standard precautions may interfere with patient health and care. A similar result was found in a study by Abhinav Singh et al. in 2011, regarding dental students in Central India. Their study showed that “The level of knowledge regarding infection control measures was poor among dental students. The attitude towards infectious control measures was positive” [7].

One of the most positive results of the study was that 93.1% of the undergraduate dental students and faculty members had been vaccinated with hepatitis B vaccine. College of Dentistry at KSU has made hepatitis B vaccination mandatory for dental students prior to clinical practice.

Percentages of students and faculty using rubber dam isolation were 60.4 and 34.4%, respectively. This could be a reflection of faculty members’ forgetting the importance of rubber dam isolation over time. The finding suggests the necessity of continuous-based infection control lectures and training.

The outcome of 3rd-year students having the highest scores of knowledge and compliance could be because it is their first clinical year in the field where the basics of infection control are overemphasized on both theoretical and practical levels. Having much heavier load in the year of graduation could explain the decrease in compliance toward infection control precautions while still obtaining a good attitude. When compared to faculty members, compliance was found to be higher in students. This may be attributed to the fact that students work under the supervision of instructors during their clinical sessions. The difference in attitude of participants may go back to the variation in person’s beliefs, thoughts, and behavioral aspect.

One of the limitations of this study was the method for assessing the practice of infection control guidelines. We could not supervise the responders’ practice and had to rely on their subjective self-assessment. Therefore, the responses might have not accurately reflected the true knowledge, attitude, and compliance. Another limitation was the absence of qualitative data that could have helped us in understanding and accessing the thoughts and feelings of the research participants. The main reason for the lack of qualitative data is the limited time available during data gathering.

Our study showed that a positive attitude and compliance toward infection control measures does not guaranty having a good level of knowledge as demonstrated in our results.

## Conclusion

The dental faculty members and students at KSU reported a good adherence to infection control precautions. The results of this study motivate for an evaluation of the taught curriculum, means of assuring compliance and an audit of whether the resources available supports the use of appropriate infection control guidelines. This evaluation could provide information of at what level changes are required in the dental curriculum. In addition, emphasizing the importance of continuous-based infection control lectures and training could help in raising the level of knowledge regarding the subject.
